# Pilot Study Results on Antibodies to the S- and N-Proteins of SARS-CoV-2 in Paired Sera from COVID-19 Patients with Varying Severity

**DOI:** 10.3390/antib12010019

**Published:** 2023-02-27

**Authors:** Yulia Desheva, Anna Lerner, Tamara Shvedova, Olga Kopteva, Polina Kudar, Irina Koroleva, Galina Leontieva, Alexander Suvorov

**Affiliations:** 1 Federal State Budgetary Scientific Institution “Institute of Experimental Medicine”, Scientific and Educational Center «Molecular Bases of Interaction of Microorganisms and Human» of the World-Class Research Center «Center for Personalized Medicine», 12, Acad. Pavlov’s str., 197022 Saint Petersburg, Russia; 2 Vsevolozhsk Clinical Interdistrict Hospital, 20, Koltushskoe Highway, 188643 Vsevolozhsk, Leningrad Region, Russia

**Keywords:** COVID-19, serum antibodies, severity, blood tests

## Abstract

In this retrospective cohort study, we investigated the formation of individual classes of antibodies to SARS-CoV-2 in archived serial sera from hospitalized patients with the medium–severe (*n* = 17) and severe COVID-19 (*n* = 11). The serum/plasma samples were studied for the presence of IgG, IgM and IgA antibodies to the recombinant S- and N-proteins of SARS-CoV-2. By the 7th day of hospitalization, an IgG increase was observed in patients both with a positive PCR test and without PCR confirmation of SARS-CoV-2 infection. Significant increases in the anti-spike IgG levels were noted only in moderate COVID-19. The four-fold increase of IgM to N-protein was obtained more often in the groups with mild and moderate infections. The IgA levels decreased during the infection to both the S- and N-proteins, and the most pronounced decrease was in the severe COVID-19 patients. The serum IgG to S-protein one week after hospitalization demonstrated a high-power relationship (r_s_ = 0.75) with the level of RBD antibodies. There was a medium strength relationship between the levels of CRP and IgG (r_s_ = 0.43). Thus, in patients with acute COVID-19, an increase in antibodies can develop as early as 1 week of hospital stay. The SARS-CoV-2 antibody conversions may confirm SARS-CoV-2 infection in PCR-negative patients.

## 1. Introduction

Currently, the global pandemic of the coronavirus disease-19 (COVID-19) caused by the severe acute respiratory syndrome coronavirus-2 (SARS-CoV-2) is ongoing [1]. Current COVID-19 vaccines have been shown to be effective against severe COVID-19, including hospitalization and death; however, there is evidence of the poor efficacy of existing vaccines against confirmed infection and symptomatic disease caused by multiple antigenic variants of the SARS-CoV-2 coronavirus [2]. The role of adaptive immunity factors in re-infection is being intensively studied. Recent studies have shown that an IgG immune response to SARS-CoV-2 infection may not develop among 2–25% of patients with COVID-19 [3]. However, patients who do not seroconvert after infection may show a strong humoral immune response upon reinfection, suggesting immunological memory formation. Although it has been shown that prior coronavirus infection may partially reduce the risk of reinfection by the Delta variant or Omicron, the duration of such protection remains unknown [3].

Clinical and laboratory data concerning the hyperinflammatory syndrome that develops in COVID-19 continue to be widely studied [4]. With the development of a ‘cytokine storm’ in COVID-19, it is proposed to take into account an increase in the serum concentration of C-reactive protein (CRP) and the neutrophil–lymphocyte ratio (NLR), which are indicators of systemic inflammatory reactions and are widely studied as predictors of the prognosis of patients with viral pneumonia, including COVID-19 [5].

At present, the mechanisms of the pathogenesis of severe coronavirus infections remain not fully characterized, despite the fact that in 2002–2003 there were cases of severe acute respiratory syndrome caused by SARS-CoV-1, as well as Middle East respiratory syndrome coronavirus (MERS-CoV) in 2012 [6]. The ways of forming protection against SARS-CoV-2 infection have not been determined, and the relationship between adaptive immunity factors and pathogenesis in COVID-19 is not well studied.

Currently, an accurate diagnosis of COVID-19 in symptomatic cases depends on using molecular analysis to detect SARS-CoV-2 RNA, and reverse transcriptase polymerase chain reaction (RT-PCR) is the ‘gold standard’. Antibody tests can provide an important complementary method for the diagnosis of COVID-19.

Of the four structural proteins of the coronavirus, the spike (S) and nucleocapsid (N) proteins are most widely expressed in viral infections and elicit an antibody response. Although the spike protein produces neutralizing antibodies, it is the least conserved among the seasonal coronavirus species. Protein N, and especially its N-terminal region, is an immunogenic and sensitive protein for monitoring seasonal coronavirus infections. In some cases, virus-specific antibodies that do not have neutralizing properties can exacerbate rather than prevent coronavirus infection due to the development of immunopathological changes. Therefore, it is very important to study which classes and subclasses of antibodies most effectively block the attachment of the virus to the receptor (angiotensin-converting enzyme 2, ACE2). The receptor-binding domain (RBD) is the key immunogenic region within the S1 protein. A competitive ELISA directed at this region of the S1 protein is likely to reflect the neutralizing properties of antibodies [7].

The objectives of our study were to assess the kinetics of serum antibodies to the S- and N-proteins of SARS-CoV-2 with varying severity of COVID-19 and establish a possible relationship with blood tests, which may be of importance for the management of COVID-19 patients and prescribing treatments.

## 2. Materials and Methods

### 2.1. Ethics Statements

A retrospective cohort study was carried out using serum/plasma samples remaining from routine clinical tests of hospitalized COVID-19 patients. The study was approved by the Local Ethics Committee of the FSBSI “IEM” (protocol 3/20 from 6 May 2020). After receiving the approval of the Ethics Committee, the sera were handed over to the researchers, none of whom had access to the personal data of patients. When analyzing the clinical data, the primary data of the patients were depersonalized and the employees did not have access to the personal data of the patients. As this was a retrospective study, informed consent was not required. Nevertheless, upon admission to the hospital, all the patients signed an informed consent, including the use of clinic samples for scientific research.

### 2.2. Study Participants and Specimens

The analyzed cohort was divided into 2 groups, depending on the severity of the course of the disease at the time of admission to the hospital. The patients were hospitalized in February–April 2020 at Vsevolozhsk Clinical Interdistrict Hospital, Leningrad Region, Russian Federation. The COVID-19 severity was estimated according to the Interim Guidelines for the Prevention, Diagnosis and Treatment of New Coronavirus Infection (COVID-19), Version 8. The medium–severe course of disease was characterized by serum CRP > 10 mg/L at the time of hospitalization, respiration rate < 30/min, dyspnea on exertion, oxygen saturation < 95%, and changes on X-ray typical of a viral lesion (lesion volume is minimal or moderate). The severe course of infection was characterized by respiration rate > 30/min, oxygen saturation < 93%, unstable hemodynamics (systolic blood pressure less than 90 mm Hg or diastolic blood pressure less than 60 mm Hg, diuresis less than 20 mL/h), changes in the lungs on X-ray typical of a viral lesion (the volume of the lesion is significant or subtotal), and arterial blood lactate > 2 mmol/L. The extremely severe course of the infection was characterized by the addition of acute respiratory distress syndrome (ARID).

A total of 28 pairs of samples were obtained on days 1–3 and 5–7 of hospital stay. Peripheral blood was collected in heparinized or ethylenediaminetetraacetic acid (EDTA) tubes. Blood samples from patients obtained before the spread of COVID-19 at the end of 2018–early 2019 were used as control samples.

After obtaining the approval of the Ethics Committee, the sera were provided for serological research in aliquots, which were stored at −20 °C until the tests were performed so that they were thawed only once.

### 2.3. Molecular Detection of SARS-CoV-2 RNA

The nasopharyngeal and pharyngeal swabs were tested by means of reverse transcriptase real-time polymerase chain reaction (rRT-PCR) at the time of hospitalization. The decision on a negative PCR test was made based on the absence of a positive signal in both the nasopharyngeal and pharyngeal swabs. Viral RNA was also determined in blood plasma samples obtained on days 1–3 of hospital stay. Viral RNA isolation from the nasopharyngeal and pharyngeal swabs as well as from the peripheral blood was performed using a nucleic acids extraction kit for clinical samples, “RealBest extraction 100” lot 226 (“Vector-Best”, Novosibirsk, Russia). Amplification of the virus nucleic acid was performed using a “RealBest RNA SARS-CoV-2” RT-qPCR system lot 7577 (“Vector-Best”, Novosibirsk, Russia) according to the manufacturer’s instructions. The test system included its own positive and negative controls and ready reaction mixture.

### 2.4. Enzyme-Linked Immunosorbent Assay (ELISA)

The blood samples were examined by ELISA for the presence of IgG, IgM and IgA antibodies specific to the SARS-CoV-2 proteins in duplicates. The test principle was based on an indirect ELISA method using microwells coated with a certain amount of the SARS-CoV-2 virus antigens. For this, Nunc MaxiSorp 96-well plates (Thermo Fisher Scientific, Waltham, MA, United States) were sensitized with a 2 µg/mL recombinant S-protein containing 496–646 amino acids (GenBank: OL447006.1) obtained in *E. Coli* as described earlier [8] or 2 µg/mL of commercial SARS-CoV-2 N-protein (AtaGenix, Wuhan, China). After 3-fold washing, serial dilutions of sera 1:4 steps were introduced into the wells of the sensitized plates. After 1.5 h of incubation at 37 °C, a washing solution was used to remove any unbound antibodies. The HRP-linked goat anti-human IgG antibody (Sigma, St. Louis, MO, USA) and rabbit-anti-human IgM or IgA (Cloud-Clone Corp., Wuhan, China) were used as conjugates. The end-point ELISA titers were expressed as the highest dilution that yielded an optical density at 450 nm (OD450) greater than the mean OD450 plus 3 standard deviations (SD) of the control wells. The average values of the control wells were determined for each dilution from 4 to 6 negative blood sera obtained before the appearance of SARS-CoV-2.

### 2.5. Concurrent ELISA Neutralization Test

A SARS-CoV-2 Surrogate Virus Neutralization Test Kit (AtaGenix, Wuhan, China), which is a kind of competitive ELISA involving the protein–protein interaction between the receptor-binding domain of the viral glycoprotein (RBD) and the surface cellular receptor ACE2, was used to detect circulating antibodies against SARS-CoV-2 that block the interaction between RBD and ACE2. The percentage of neutralization was calculated based on OD450 according to the manufacturer’s instructions.

### 2.6. Laboratory Data

The serum concentration of C-reactive protein (CRP) was determined by the turbodimetric method using BioSystems reagents (BioSystems, Barcelona, Spain). The neutrophil/lymphocyte ratio (NLR) was calculated based on clinical blood test data using the following formula: absolute neutrophil count/absolute lymphocyte count.

### 2.7. Statistical Analysis

The statistical data processing was performed using the Statistica 12.0 software package (StatSoft, Inc. Tulsa, OK, USA) and the graphics data were generated using Prism 8 (GraphPad software, San Diego, CA, USA). The data were normalized using the mean normalization method (Z-normalization). The medians (Me) and lower and upper quartiles (Q1; Q3) were calculated and used to present the group averages. The antibody titers were expressed as the geometric mean titers (GMT) or means plus standard deviation (SD). The comparisons of two independent groups were made using a nonparametric test, namely the Mann–Whitney test. The comparisons of two dependent variables were performed using the Wilcoxon matched pairs test. For the nominal data, Fisher’s exact 2-tailed test was used. The presence of a statistical relationship between the variables was estimated via a correlation analysis in Python 3 using the Pandas library ‘Corr’ function using Spearman’s method. The *p*-value < 0.05 was considered to be statistically significant.

### 2.8. Study Limitations

This study had several limitations. As this was a retrospective study, the researchers did not have access to some personal information of inpatients, some in a serious condition. We had the opportunity to associate each of the analyzed samples with varying degrees of severity of the disease according to specific signs determined by the recommendations of the Ministry of Health of the Russian Federation, as indicated in the Materials and Methods. From the previous anamnesis, it was known that none of the patients studied were vaccinated with the Sputnik V vaccine or other COVID-19 vaccines, for the reason that the study was carried out using blood sera obtained in March–April 2020 when no COVID-19 vaccines were used.

## 3. Results

In this retrospective cohort study, we evaluated pared serum/plasma samples obtained from 28 patients with medium–severe and severe coronavirus infection caused by the SARS-CoV-2 virus, who were hospitalized in the Leningrad Region Vsevolozhsk Clinical Interdistrict Hospital during February–April 2020, including 28 paired samples. Table 1 presents some demographic and clinical parameters among the two groups of patients.

As can be seen from Table 1, in general, the rates did not differ significantly between the groups of patients with moderate and severe COVID-19. The exception was that a positive PCR test for SARS-CoV-2 was detected more often in medium–severe disease (*p* = 0.03) compared to severe COVID-19. Viremia, determined by the detection of viral RNA in the blood by PCR, was more common among patients with severe infection, although the differences were not statistically significant (*p* = 0.06). It is noteworthy that viremia was detected in three PCR-negative COVID-19 patients and in two patients with PCR-confirmed COVID-19.

**Figure 1:**
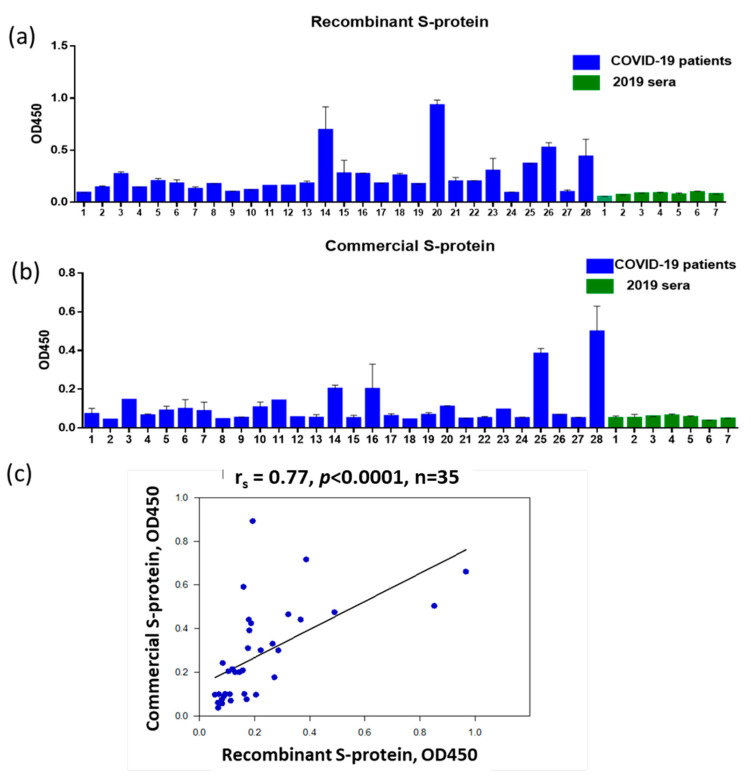
Detection of IgG antibodies to SARS-CoV-2 in serum samples (1:100 dilution). The abscissa axis shows the serial numbers of the studied sera of 28 COVID-19 patients collected on days 1–3 of hospitalization (blue bars) and the serial numbers of 7 sera collected from healthy persons examined in 2019 (green bars). All the blood samples were tested in duplicates. (**a**) ELISA read data obtained using recombinant S-protein containing 496–646 amino acids (GenBank: OL447006.1). (**b**) ELISA read data obtained using commercial S-protein (AtaGenix, Wuhan, China). (**c**) Correlation analysis of mean OD_450_ values obtained using recombinant S-protein or commercial S-protein.

To assess the serum immunoglobulins to the S-protein of SARS-CoV-2 via ELISA, we used recombinant S-protein containing 496–646 amino acids based on the N-terminal part of the S1 protein with the inclusion of the RDB region (GenBank: OL447006.1) (Figure 1a). A bioinformatics analysis of the S-protein sequence obtained from the NCBI database revealed an extended immunodominant B-cell linear epitope of 52 amino acids NFNGLTGTGVLTESNKKFLPFQQFGRDIADTTDAVRDPQTLEILDITPCSF in the area of close proximity to RBD. We also tested the sera for IgG to SARS-CoV-2 S-protein using a commercial S-protein (AtaGenix, Wuhan, China) (Figure 1b). Of the 28 patients screened at hospital admission, 20 donors were IgG positive based on the greater than mean OD450 plus 3 standard deviations (SD) of negative sera for panel A and 16 donors were seropositive for panel B. The differences were not statistically significant (*p* = 0.4, Fisher’s exact test). A high level of correlation between the ELISA read data obtained using recombinant S-protein and commercial S-protein was shown (Figure 1c). Taken together, these data allow us to conclude that the recombinant S-protein we have developed is suitable for assessing antibodies in paired sera from patients with COVID-19.

**Figure 2:**
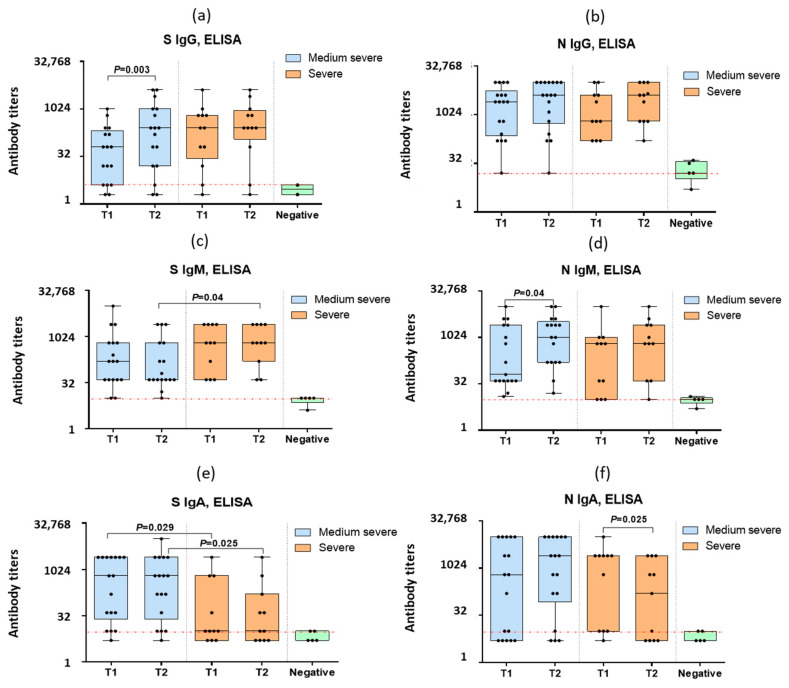
(**a**–**f**) Serum immunoglobulins G, M and A in paired sera of 28 patients with medium–severe COVID-19 (*n* = 17) and severe COVID-19 (*n* = 11). The box extends from the 25th to 75th percentiles. The line in the middle of the box is plotted at the median. The whiskers go down to the smallest value and up to the largest. T1—blood samples obtained on days 1–3 of hospitalization, T2—sera obtained on days 6–7 of a hospital stay. “Negative”—means negative control sera obtained at the end of 2019–early 2020 from patients who were not in contact with SARS (*n* = 5). The red lines indicate the sensitivity threshold of the method, which was the starting dilution of the tested samples—1:4 or 1:10. Black dots indicate individual antibody titers.

For the further study of the anti-spike IgG, IgM and IgA antibodies in the blood serum of the COVID-19 patients, the above-mentioned recombinant S-protein was used. In the paired sera of the COVID-19 patients, detectable levels of antibodies to the S- and N-proteins of SARS-CoV-2 were obtained (Figure 2).

Statistically significant increases in the mean anti-spike IgG levels were noted in the case of medium–severe COVID-19 but not during severe infection (Figure 2a). The IgG levels to N-protein increased in both groups (Figure 2b), although the differences were not statistically significant. In contrast to the IgG levels, the IgM levels to spike decreased with a moderate infection (Figure 2c) so that the level of anti-spike IgM in the repeated sera in moderate COVID-19 was significantly lower than in severe COVID-19 (Figure 2c). The IgM levels to N-protein increased in the medium–severe COVID-19 patients (Figure 2d). The spike IgA levels were significantly lower among the severe COVID-19 patients compared to those with moderate infection (Figure 2e). The IgA levels to N-protein of SARS-CoV-2 significantly decreased in severe COVID-19 (Figure 2f).

It can be noted that an IgG antibody increase was observed in patients both with a positive PCR test and without PCR confirmation of SARS-CoV-2 infection (Figure 2).

**Figure 3:**
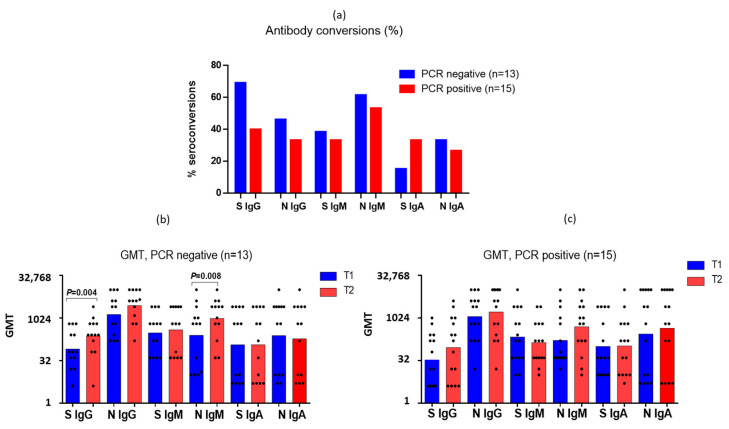
Analysis of antibody levels to SARS-CoV-2 proteins among patients with PCR-negative and PCR-positive nasopharyngeal and pharyngeal swab tests by real-time PCR. T1—blood samples obtained on days 1–3 of hospitalization, T2—sera obtained on days 6–7 of a hospital stay. (**a**) The proportion of individuals with a 4-fold or more increase in antibodies. (**b**) Geometric mean titers (GMTs) of antibodies among PCR-negative patients. Black dots indicate individual antibody titers. (**c**) GMTs of antibodies among PCR-positive patients. Black dots indicate individual antibody titers.

Interestingly, the number of seroconversions determined by a four-fold or more increase in the antibody levels among the PCR-positive patients was slightly less than among the PCR-negative ones (Figure 3a). The initial mean antibody levels to the S- and N-proteins of SARS-CoV-2 did not differ significantly among the PCR-negative and PCR-positive patients (Figure 3b,c). It was shown that among both the PCR-positive and PCR-negative patients there was an increase in the IgG titers to SARS-CoV-2 S-protein, but only among the PCR-negative patients was this increase statistically significant (Figure 3b). The same was shown for IgM to N-protein (Figure 3b,c). At the same time, in the PCR-negative patients, a slight decrease in anti-N IgA antibodies was observed (Figure 3b), in contrast to the seropositive patients (Figure 3c). The anti-S IgM decreased slightly in the seropositive patients. The obtained data on the seroconversions in the PCR-negative patients suggest that the measurement of antibodies in paired blood sera can serve as an additional diagnostic criterion in cases where the virus is not detected by PCR.

**Figure 4:**
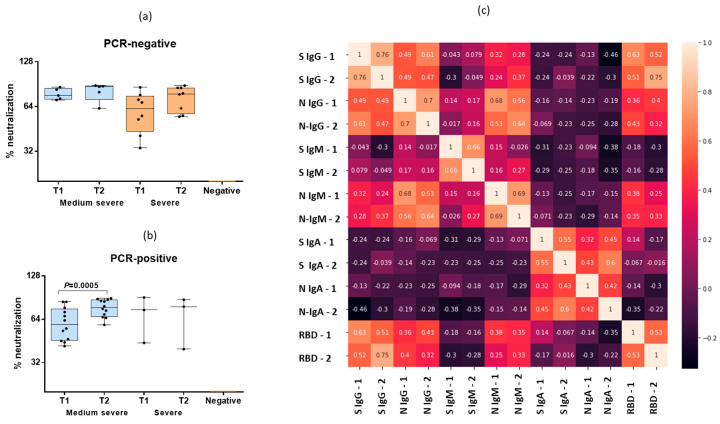
Analysis of RBD-blocking antibodies. (**a**) Percentage of neutralization using a competing ELISA in paired sera of PCR-negative COVID-19 patients (*n* = 13). T1—blood samples obtained on days 1–3 of hospitalization, T2—sera obtained on days 6–7 of a hospital stay. “Negative”—means negative control sera obtained at the end of 2019–early 2020 from patients who were not in contact with SARS (*n* = 5). (**b**) Percentage of neutralization using a competing ELISA in paired sera of PCR-positive COVID-19 patients (*n* = 15). T1—blood samples obtained on days 1–3 of hospitalization, T2—sera obtained on days 6–7 of a hospital stay. “Negative”—means negative control sera obtained at the end of 2019–early 2020 from patients who were not in contact with SARS-CoV-2 (*n* = 5). (**c**) Spearman correlations of various classes of antibodies in paired sera of patients with COVID-19 (*n* = 28). Black dots indicate individual antibody titers.

An increase in the RBD antibody levels was observed among both the PCR-positive and PCR-negative COVID-19 patients (Figure 4a,b), although the antibody levels increased statistically significantly only in the PCR-positive patients with moderate COVID-19 infection (Figure 4b). It was shown that only the IgG to S-protein in the blood samples one week after hospitalization demonstrated a high-power relationship (r_s_ = 0.75) with the level of RBD antibodies in the same serum samples. A medium-strength relationship with the RBD antibodies was found in the case of the IgG to S-protein in the blood samples sera at admission to hospital (r_s_ = 0.63). Thus, only the IgG to S-protein demonstrated strong correlation with the RBD antibodies. These data once again confirm the relevance of the recombinant S-protein containing 496–646 amino acids for the detection of SARS-CoV-2-specific antibodies. A medium-strength relationship was found in the case of IgG and IgM to the N-protein (r_s_ = 0.68 in the first sera and r_s_ = 0.64 in sera taken during re-examination). Another medium-strength relationship was found between IgG and the S- and N-proteins of SARS-CoV-2, but much weaker (r_s_ = 0.47–0.49) (Figure 4c).

**Figure 5:**
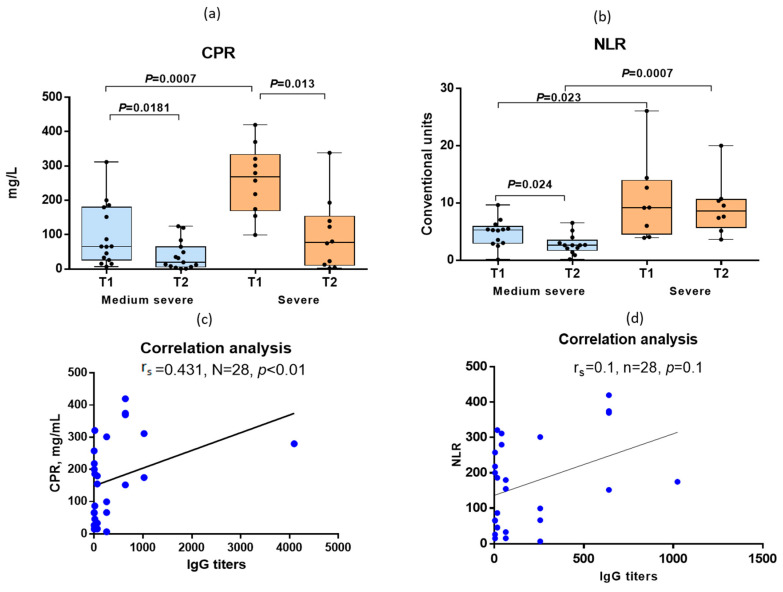
Dynamics of blood tests in COVID-19 patients. (**a**,**b**) Mean levels of CRP and NLR (mean, SD) in medium–severe COVID-19 patients and severe COVID-19 patients. T1—blood samples obtained on days 1–3 of hospitalization, T2—sera obtained on days 6–7 of a hospital stay. (**c**) Correlation analysis of IgG titers and CRP levels in sera at the time of hospitalization. (**d**) Correlation analysis of IgG titers and NLR levels in sera at the time of hospitalization. Black dots indicate individual antibody titers.

While CRP was used as one of the criteria for determining the severity of infection, the NLR levels were also statistically significantly different between the medium–severe and severe COVID-19 patients (Figure 5a,b). A medium-strength relationship was noted between the content of CRP and the levels of IgG to the S-protein of SARS-CoV-2 in the same blood sera (r_s_ = 0.431) (Figure 5c). At the same time, we did not find any relationship between the NLR values and antibody titers (r_s_ = 0.1) (Figure 5d).

Thus, examination of patients with acute COVID-19 has shown that SARS-CoV-2 antibody conversions may additionally confirm SARS-CoV-2 infection in PCR-negative patients. Laboratory values with the severe course of COVID-19 were characterized not only by a significant increase in serum CRP but also NLR compared with medium–severe COVID-19. The level of viremia determined by detection of viral RNA in the blood using PCR was also higher with severe COVID-19 compared with moderate infection.

## 4. Discussion

The identification of SARS-CoV-2 genetic material using PCR is one of the main criteria for COVID-19 diagnosis. At a late stage of the disease, the viral RNA may not be detected by PCR. In our study, the RNA of SARS-CoV-2 was detected less frequently among COVID-19 patients with a severe course compared with patients with a moderate form of the disease. A possible explanation for this is the replication of the virus in the deeper parts of the respiratory tract during severe infection, since the PCR swab was collected from the nose and throat.

Despite the fact that the WHO does not recommend the determination of antibodies as confirmation of COVID-19 [9], a number of studies show that the estimation of IgG and IgM can confirm the diagnosis to 83.9% in cases where SARS-CoV-2 is not detected by PCR [7]. A single blood test for antibodies is not very informative; studies of serial clinical samples obtained from the same patients are of interest. Various criteria have been proposed to predict the severity of COVID-19, including levels of virus-specific antibodies, neutrophil counts, and serum cytokine levels. The magnitude of early antibody responses may be indicative of the severity of infection, because higher IgM and IgG antibody titers are associated with more severe disease [10]. Moreover, some researchers believe that antibodies to the N-protein of the SARS-CoV-2 may be an indicator of a more severe course of COVID-19, as they are associated with a more frequent development of viremia [11]. Our study showed an increase in IgG and IgM to S-protein in severe COVID-19 compared with moderate infection, and the level of viremia was also higher in the severe course of coronavirus infection.

Since the days from the onset of illness differed significantly between the patients with moderate and severe infections, as shown in Table 1, patients with moderate infections were examined, on average, later after illness onset and may have already begun to rise in antibodies. However, there were statistically significant increases in serum anti-spike IgG in the moderately severe patients compared to the patients with severe infection (Figure 2a). The same applies to IgM antibodies to N-protein (Figure 2d). The only class of antibodies that, upon admission to the hospital, was statistically significantly higher in the moderate patients compared to the severe ones is anti-spike IgA (Figure 2a).

Previous SARS-CoV-2 studies have shown that IgM antibodies can be detected as early as three days after infection, providing the first line of humoral immunity defense, while high-affinity IgG antibodies are produced after seven days [12,13]. Previously, it has been shown that high titers of IgG antibodies detected by ELISA positively correlate with neutralizing antibodies [14]. Neutralizing antibodies are a major factor in an effective human immune response to many pathogens, but their role in coronavirus infection is unclear. Neutralizing antibodies have the ability to block the virus, which is binding to the ACE2 receptor in human cells. Neutralizing antibodies make it possible to eliminate the action of invading microorganisms, and their activity is generated by proteins located on the surface of viruses with which they bind to “block” infection. Various studies on neutralizing antibodies against SARS-CoV-2 show that these antibodies appear about two weeks after the start of infection, and their peak activity occurs at 4–6 weeks [10]. However, it has not yet been confirmed whether all patients produce neutralizing antibodies, what factors determine their appearance and activity, and whether their levels of neutralization are always sufficient to provide protection, since the levels vary greatly and are not detected in 10–30% of patients, and from 50 to 70% of patients have antibodies whose neutralizing activity is average or medium–low, and only a minority of patients, from 1 to 5%, have high neutralization titers [11].

We have compared the levels of S- and N-specific IgG, IgM and IgA antibodies with antibodies to the RBD site analyzed using competitive ELISA. This test to some extent can give an idea of the neutralizing properties of antibodies in the blood. It was shown that only IgG to S-protein after a week of hospital stay correlated with the latter test. Previously, it was shown that RBD-specific antibodies, and especially antibodies to N-terminal region of S1, are most important in neutralizing the virus, as they prevent the virus from attaching to the cell [15,16].

The role of the virus-specific IgA antibody response in SARS-CoV-2-induced lung injury remains to be clearly defined [17]. It has been shown that there is a connection between IgA deficiency and repeated upper or lower respiratory tract infections. Some studies suggest that dimeric IgA may be more effective in neutralizing the SARS-CoV-2 virus than IgG [18], especially in the early stages of infection [19]. Our study showed that the serum IgA levels not only were lower in patients with severe infection compared to medium–severe COVID-19 patients, but also decreased with the course of the disease. Perhaps this serological test could serve as one of the prognostic criteria for the severity of the disease.

Several studies have shown that after asymptomatic or mild COVID-19, the presence of anti-spike protein IgG antibodies is associated with a significantly reduced risk of reinfection within 9 months. It has been shown that protective immunity mediated by T-cells in a significant proportion of cases is associated with the development of anti-spike IgG [20]. Immunity acquired after infection has been shown to have the same protective effect against re-infection with SARS-CoV-2 as vaccination with the vaccine [21].

CRP is the main laboratory marker of the activity of the pathological process in the lungs. An increase in CRP correlates with the volume of lung tissue damage and is the basis for the initiation of anti-inflammatory therapy. CRP is an early marker of the inflammatory process in the lungs that occurs during coronavirus infection, and its level is directly related to the severity of the disease [22]. Normally, the level of CRP in the blood does not exceed 5 mg/L. According to studies of clinical indicators in patients with COVID-19, there was a significant increase in the level of CRP (on average, from 20 to 50 mg/L). In patients with severe COVID-19, the CRP values were significantly higher compared to patients with a mild form of the disease [23]. Previously, it was noted that the probability of developing a severe course of disease increases by 5% for each unit of increase in the level of CRP in patients with COVID-19 [23].

Changes in the concentration of CRP observed in COVID-19 also reflect not only inflammatory processes, but also indicate the presence and course of bacterial coinfection. The results of numerous studies show that there is a direct correlation between the levels of CRP in patients with COVID-19 and their respective outcomes in terms of death and length of hospital stay. CRP can be used as a prognostic marker in patients with COVID-19. Clinically significantly elevated CRP levels may be an indication for more aggressive treatment of COVID-19. In patients who died from COVID-19, the level of CRP was about 10 times higher than in those who recovered [24]. Moreover, in severe infections, CRP levels were associated with higher anti-spike IgG antibody titers [23,24,25,26]. In our study, the CRP levels moderately correlated with anti-S IgG but not at all with anti-N IgG, despite the high anti-N IgG titers. The higher level of antibodies to the N-protein can be explained by the fact that, as shown earlier, IgG antibodies and total Ig to N-protein are formed on average 2 days earlier than anti-S IgG [27]. The NLR levels are also seen as an important indicator of inflammation in COVID-19. Previously, it was shown that the average value of NLR with a mild course of the disease was 5.6, and with a severe course it was an average of 9.2, and this difference was statistically significant. It was also noted that the NLR value had a positive correlation with the assessment of the severity of pneumonia, length of hospital stay, and CRP and D-dimer levels [28]. In our study, the levels of not only CRP but also NLR were statistically significantly different between moderate and severe COVID-19. Moreover, the fact that the level of CRP correlated with the level of IgG confirms the need to study the Fc-mediated reactions of this class of immunoglobulins [29]. Previously, it was shown that IgG to the RBD of the S-protein of MERS coronavirus was able to enhance virus entry into cells through the Fc fragment of the antibody attached to cell Fc receptor (FcR) [30]. However, with COVID-19, this needs to be studied in further research. Therefore, further studies of all the components of innate and adaptive immunity in COVID-19 will contribute to a better understanding of these issues.

## 5. Conclusions

The developed recombinant SARS-CoV-2 S-protein is suitable for the detection of antibodies in blood serum. Serological studies may additionally confirm SARS-CoV-2 infection when the virus is not detected by PCR. In our study, the detection of serum IgG to the SARS-CoV-2 S-protein most closely corresponded to the anti-RBD activity of the studied blood samples. In patients with acute COVID-19, an increase in antibodies was registered as early as 1 week of hospital stay. There was a medium-strength relationship between the level of CRP and anti-S IgG (r_s_ = 0.43), which may indicate a relationship between innate and adaptive immunity factors in COVID-19.

## Figures and Tables

**Table 1:** COVID-19 patient characteristics.

Parameters	Patient Categories		*p* =
	Group 1—Medium Severe (*n* = 17)	Group 2—Severe (*n* = 11)	
Age, Me (Q25;Q75)	65.3 (58.0;70.5)	63.0 (51.0;76.0)	0.77
Male	10 (58.8%)	7 (63.6%)	0.59
Female	7 (41.2%)	4 (36.4%)	0.59
Days from onset of disease, Me (Q25;Q75)	8 (5.5;11.5)	4 (3.0;8.0)	0.013
Positive PCR-test for SARS-CoV-2 on day of admission to hospital	12 (70.6%)	3 (27.3%)	0.03
Virusemia (positive serum PCR-test)	1 (5.8%)	4 (36.3%)	0.062
Time of taking the 2nd blood sample, Me (Q25;Q75)	4.0 (4.0;4.5)	4.0 (4.0;4.5)	0.28
Lethal outcome	0 (0%)	3 (27.3%)	0.05

## Data Availability

All data are contained in the manuscript.
